# The Practical Treatment of True Pyorrhea Alveolaris

**Published:** 1910-10-15

**Authors:** J. P. Buckley

**Affiliations:** Chicago, Ills.


### THE PRACTICAL TREATMENT OF TRUE PYORRHEA ALVEOLARIS.

BY J. P. BUCKLEY, PH.G., D.D.S., CHICAGO, ILLS.

It is not my purpose to discuss the pathology of pyorrhea alveolaris, and I shall make no attempt to settle the much-mooted question as to whether the disease is of purely local origin or whether it is a local manifestation of some constitutional disease, I shall endeavor to express in definite terms my idea of how the disorder should be treated from the practical viewpoint. It is therefore to this phase of the subject that I desire to direct your attention. I want to say however, at the very outset, that I realize that the best way to treat any disease is to ascertain the cause and try to prevent its development.

### TRUE PYORRHEA ALVEOLARIS AND ITS SYMPTOMS.

It is my opinion that a mistake has been made by the profession generally in the past in grouping too many of the diseases of the gum and the pericemental and alveolar tissues under one heading—calling them all pyorrhea. When I speak of pyorrhea alveolaris in this paper I mean true pyorrhea, and exclude all of those perverted conditions of the gum which can be restored to normal by a thorough prophylactic treatment. In true pyorrhea alveolaris I am here going to include those aggravated cases which present to every practicing dentist, the treatment of which calls for more than the simple removal of the deposits from under the gum margin and the polishing of the tooth-surfaces, however thoroughly it may be done. In these cases, as a rule, there is no gingivitis, interstitial or otherwise; the gum, in fact, is rather anemic, having a tendency to recede from the necks of the affected teeth. The pericemental membrane is being rapidly destroyed; the alveolar process is resorbed—virtually is melting away; the pockets are deep and variously located around a single root of a tooth, and frequently between the roots of multi-rooted teeth. Pus is generally present and exudes from the pockets upon pressure; hard deposits may or may not be present. There is a progressive loosening of the teeth, and unless the disease is checked they ultimately drop out.

### THE DOUBTFUL ETIOLOGY OF PYORRHEA ALVEOLARIS.

The true cause of this kind of pyorrhea—if there be more than one kind—has never been satisfactorily determined. It may be a bacterium, but as yet no specific germ has been isolated and proved to be the exciting agent, though the presence of various pathogenic germs has been scientifically demonstrated, as is evidenced also clinically by the pus formation. The cause of this condition cannot be wholly neglect on the part of the patient, for the disease occurs at times in the mouths of patients who take unusually good care of their teeth. The neglect in far too many instances can rather be traced to the family dentist, who either failed to recognize and correct the disease in its incipiency, or on recognizing it informed the patient that nothing could be done, and advised extraction as the only means of affecting a cure. It is a blot on the fair escutcheon of dentistry that so many teeth have thus been neglected and ultimately lost because of this seeming indifference of dentists. There are two sides to this question, however, for on the other hand we find men who claim to be able to cure every tooth affected with pyorrhea, however badly, and this frequently results in as much discouragement to the conscientious dentist who refers his pyorrheal patients to the so-called pyorrhea specialists as it does to the patients themselves. There has been some slight excuse, therefore, for this seeming indifference.

### THE PATIENT'S CO-OPERATION.

When cases of this kind present for treatment the first important consideration is to impress upon the mind of the patient the fact that in order to obtain satisfactory and permanent results, it will be necessary to have his hearty co-operation, and he should follow instructions closely. This is exceedingly important, for there is perhaps no operation which dentists are called upon to perform wherein the patients' confidence and especially their co-operation are such vital factors, so far as permanent results are concerned, as in the treatment of the condition under consideration; and it is truly surprising what good results can be obtained by the conscientious and persistent efforts of both patient and dentist.

### INDICATION FOR EXTRACTION BEFORE PYORRHEA TREATMENT.

Before undertaking the treatment of a case, then, a thorough examination should be made, and there should be a distinct understanding as to what may reasonably be expected. The discerning patient will quite naturally want to know if his case can be cured. The word "cure" here can have but a relative meaning, and an explanation should be made in regard to what a so-called cure includes. It should not mean, in my opinion, that the lost tissues, *i. e.* gum, pericemental and alveolar tissues, can be restored. It should mean, however, that the teeth which you undertake to treat can ultimately be made healthy, comfortable, and useful, so far as mastication is concerned; and any tooth which cannot be restored to this condition had better be extracted at once. This is the time to extract the hopelessly affected teeth, not after they have been treated for six months or a year. Nothing is more discouraging to patients than to have to lose a tooth after extended treatment. It has a tendency to make them question your ability to save any of the affected teeth. The experienced dentist should know from the condition he finds whether or not it is best to save the tooth. In this connection I desire to state that there are usually many hopelessly affected teeth in the mouths of pyorrheal patients.

### PROGNOSIS OF CURE.

While the presence of pus, which is constantly being mixed and swallowed with the saliva and food, causing digestive disturbances and perhaps slow toxic poisoning of the general system, is of greater importance as far as health is concerned than is the looseness of the teeth in the jaw, patients, as a rule, are more interested in the latter phase of the question, and are always anxious to know whether the now loosened teeth will tighten and become firm after the treatment. This depends entirely upon the case at hand and the mechanical treatment involved. In the past, too much dependence has been placed on drugs, and too often also it has been left to nature to tighten the loosened teeth, when that should not be expected, on account of the extensive loss of tissues. The conscientious dentist who makes extravagant statements in this respect will have much cause for regret, as well as give much disappointment to his patient. I desire to speak emphatically on this point, for herein often lies the greatest scource of disappointment; yet I dare say that each of you has been surprised, at times, how loosened pyorrheal teeth will tighten in the jaw under proper treatment without mechanical assistance other than the correction of the occlusion; but nevertheless, not too much should be promised in this respect. Unless the end of the root is resorbed, leaving it roughened, the pus formation can be checked and the gum can be made to resume a healthy appearance, but many times the teeth, without a permanent mechanical aid, will not materially tighten, though otherwise they are perfectly healthy and comfortable. The usefulness of such teeth, of course, is impaired by their looseness.

It is therefore important that this be properly understood at the beginning, for it prevents disappointment, it stimulates confidence in the operator's ability to treat the case, and it evidences the conscientiousness with which he works.

No dentist should undertake the treatment of a case of true pyorrhea unless he thoroughly understands the case and its requirements, and is equipped to render the best possible service, considering always the personal equation, and then only with the understanding that both he and the patient are to work in harmony, both exerting their best efforts to eradicate the disease. It is far better not to undertake the treatment of a case than to have it end in disappointment. This is not said with the idea of discouraging the general dental practitioner, but rather to stimulate all of us to do better work.

The practical treatment is threefold, surgical, medicinal, and mechanical, and each of these phases will be discussed here under its respective heading.

### SURGICAL TREATMENT.

*Instruments used.* The surgical treatment includes the scaling of the teeth and the curetment of such parts as is necessary. To do this it is essential that we use such instruments as will accomplish the end in a definite and certain manner. The instruments which I use are the Logan-Buckley set. By the use of this set of twenty-five instruments, so devised as to reach all surfaces of the tooth-structure, the scaling can be done in a thorough and systematic manner, and with a minimum amount of injury to the soft tissues. If deposits are present they must be removed, and if absent, the pockets, including the affected tooth-surface and alveolar process, must be thoroughly curetted.

*Antiseptic preparation of the mouth.* Before beginning the scaling and curetting process, the mouth should be sprayed or rinsed with a warm antiseptic solution. I use for this purpose cinnamon water, to which about 2 per cent. of alcohol is added. This is a pleasant and agreeable solution. A glassful should always be in a convenient place, so that it may be used frequently with a strong water syringe to clear the field of operation. It is well to color the solution pink or red with tincture of cudbear, as this prevents the patient from seeing the blood which may follow the operation; although in this class of cases, if the proper instruments are used, there is little necessity of causing much hemorrhage.

*Procedure of scaling.* The scaling should be begun on some particular tooth in the mouth, and this tooth should not be left until it is thoroughly scaled. This can only be done by following some definite system. I begin with instrument No. 1 of the Logan-Buckley set on the mesio-buccal surface of the upper right third molar, and with the six instruments (Nos. 1 to 6 inclusive) designed for this purpose one can work completely around the tooth in a methodical manner. If at any point a pocket is found too deep to be reached with these instruments, the counterpart of each, differing only by a narrower point and the exaggeration in form, is found in Nos. 16 to 21 inclusive. For example, No. 16 is the exaggerated form of No. 1, and so on throughout the entire six instruments. For the lower teeth the positions are exactly reversed. With one or the other of these groups of six instruments the molars and bicuspids can be scaled, except occasionally where deep pockets are found between the roots of molars and on the lingual surfaces of divergent lingual roots of upper molars. For these exceptional cases Nos. 9 and 10 are designed for right and left use, and are employed for scaling and curetting between the roots of molars, and Nos. 24 and 25 for right and left use on the lingual surface of divergent lingual roots of upper molars. No. 23 is for the distal surface of posterior teeth. In scaling the anterior teeth No. 7 is designed for the lingual and No. 8 for the labial surface. In cases where there is a deep lingual pocket which cannot be reached with No. 7, No. 22, the exaggerated form, can be used. No. 11 is a push instrument used in removing deposit from between crowded and irregular teeth. No. 15 is a general hook used for removing bulky deposit, wherever found, before the more delicate instruments are used. Nos. 12, 13, and

14 are prophylactic instruments which can be used with either a push or pull movement, and will be found useful in removing superficial decay and in polishing rough surfaces.

I have thus briefly described the use of the Logan-Buckley set of scalers, for while the system is not complicated, nevertheless it is essential that the operator should know exactly where each instrument should be used, so that the work may progress in a definite manner.

*Sterilization of scaling instruments.* After an instrument has been used, its point at least should be immersed in an antiseptic solution during the entire time the scaling is being done. To accomplish this, it is necessary to have a tray designed in such a manner as to hold the instruments in their proper place. It is a principle in surgery to avoid the presence of germs rather than to endeavor to kill them by the use of strong disinfectants during the operation. The instruments, therefore, should be sterile before they are used, thus avoiding the necessity for using strong disinfectant solutions in the tray. Such solutions would either act upon the metal, or would taste badly when the instrument is placed in the patient's mouth; for the proper instrument should be taken directly from the rack of the tray and used in the mouth. Therefore all that is necessary is to have a pleasant antiseptic solution in the tray. I use a 20 per cent. solution of phenol compound in glycerin, and of this about 10 minims is added to about one-half pint of sterile water, which is poured into the tray. This solution also may be colored with tincture of cudbear.

*Duration of scaling operation and prophylactic treatment.* The scaling operation should generally be limited in time to one hour, and the scaling process should be stopped in sufficient time to permit a thorough prophylactic treatment with orange-wood, pumice, and tape, according to the method of D. D. Smith, before applying the medicinal treatment.

*Indications for extirpation of pulps.* The question of removing the pulps from the badly affected teeth is an important one, and should not be overlooked. There are at least three indications for the removal of the pulp in these cases, viz, first, where the pockets are deep and the infection in the apical area has so lowered the vitality of the pulp that ultimate death is the inevitable result; second, where the unnatural exposure of tooth-surface results in hypersensitiveness to the degree of constantly annoying the patient, and third, where the adjustment of a permanent retaining appliance is necessary.

It has been clinically observed that the removal of the pulp from a badly affected pyorrheal tooth is followed by a more rapid response to the local treatment of the disease, and many have advanced the theory that this result is produced by directing the entire circulation of the tooth to the sluggish pericemental membrane. On other occasions I have expressed a similar opinion myself, but the idea is erroneous. Noyes, Broomell, and others have shown that the pericemental menbrane normally is well supplied with bloodvessels, and therefore extirpation of the pulp would not materially affect the circulation to this membrane.

*Amputation of roots.* Frequently we find cases where a single root of a multi-rooted tooth is practically denuded of its pericemental attachment the remaining roots being in fairly good condition. The affected root in these cases should be completely excised. This of course necessitates the removal first of the pulp and filling the root. If two roots are thus affected, the tooth had better be extracted.

*Local anesthesia contra-indicated.* In connection with this surgical treatment I should mention the use of local anesthetics, for it is the practice of many dentists to anesthetize the parts operated upon. I would not advise the use of local anesthetic agents, for a further poisoning of the tissues by actual injection, when they are already in a low state of vitality, is contra-indicated, and the slight anesthesia otherwise induced is too often obtained only at the expense of neatly as much pain as the careful scaling and curetting with the proper instruments will produce.

The tissues, not being anesthetized, serve as a guide for the careful operator in locating and following the pockets to the end, when the surgical treatment can be accomplished with a minimum amount of injury to the soft parts involved.

### MEDICAL TREATMENT.

*An astringent remedy.* When the surgical treatment is completed—and the teeth operated upon have been thoroughly polished, the mouth should again be sprayed with a warm antiseptic solution, forcing it between the teeth and under the gum margin in order to mechanically remove any loose deposits or pumice which may have been left from the polishing. The parts should then be kept dry by the use of Japanese bibulous paper or cottonoid rolls, and an application of some astringent remedy should be made. For this purpose I use the following:

| | | |
|---|---|---|
| ℞—Potassii iodidi, | ʒij | |
| Iodi (crys.), | ʒijss | |
| Zinci phenol-sulfonatis, | ʒij | |
| Aquae, | fʒvj | |
| Glycerini, | fʒiijss. | M. |

Sig.—Apply freely to the gums.

*The patient's duty of brushing and massaging his teeth and gums.* After this remedy has been applied for a moment, the mouth should again be sprayed or rinsed. The patient should be instructed to properly brush, massage, and care for his mouth and teeth generally. Prescriptions should then be written for an antiseptic mouth-wash and tooth-powder or paste, and the patient be dismisseid until the next sitting. In instructing the patient relative to brushing the teeth, it should be emphasized that the gums should be vigorously brushed as well as the teeth, thus securing the benefit of the massage. It should be remembered here that both patient and dentist are confronted with a serious problem, and therefore the instructions should be explicit, and the progress of the case be watched closely.

Iodin is a penetrating and stimulating agent, and is especially efficacious in these bone affections. If the surgical treatment has been thorough, the case generally yields nicely. The astringent remedy may be applied at first every two or three days. The intervals for the subsequent applications should gradually be lengthened, and in about two or three weeks the case should be ready for the patient's own care.

*Cauterization with phenol-sulfonic acid in stubborn cases.* It is occasionally necessary in a stubborn pus pocket to resort to stronger remedies and to thoroughly cauterize the area involved. For this purpose I use a 25 or 50 per cent. solution of phenol-sulfonic acid. I have recently succeeded in having this agent prepared in such a manner that practically all of the phenol and sulfuric acid used in the manufacture of phenol-sulfonic acid is in a combined state. This makes a very efficacious remedy. As a rule the use of acids in the treatment of pyorrhea alveolaris is contra- indicated because of the extreme hypersensitivity which they produce.

*Amputation of root-apices or extraction in persistent cases.* When after the above treatment the pus still persists, we can suspect resorption of the root in the apical area, leaving sharp, needle-like points, which prevent healing. In this case it is necessary to excise the root-end or extract the tooth. When the root is completely or partly excised, the cavity thus formed may be filled with Beck's cerate of bismuth subnitrate. This stimulates the affected part and prevents reinfection from the oral cavity.

*Replantation.* Some writers advocate replantation in these cases, but my experience in this has not been satisfactory. For permanent results it is absolutely necessary that the replanted tooth be held firmly in position by some retaining appliance, and it has been my experience generally that extraction and the insertion of an artificial substitute is far more sanitary and satisfactory. It is not a question here of what a dentist can do by his ingenuity and skill, but rather of what is bestfor the patient.

*Systemic treatment.* There are surely cases of true pyorrhea alveolaris where the local condition in the mouth seems to be aggravated, if not caused, by some systemic disease. It is the plain duty of the dentist here to collaborate with the family physician. In fact, when we are certain of our diagnosis of systemic disturbances, it is almost useless to undertake the local treatment unless measures are simultaneously instituted for the correction of the systemic disease.

*Mouth-washes and tooth-pastes.* The general prescribing of mouth-washes which contain astringent drugs, especially the zinc salts, in the treatment of pyorrhea alveolaris, in my opinion is wrong. Certain tissues of the mouth need to be constringed and stimulated to healthy activity, but the entire mucous membrane surely does not need to be so treated. In a series of experiments on dogs, Cook has shown that the continuous use of astringent mouth-washes interferes for hours with the action of the ptyalin upon starchy foods. The astringent remedy should be applied by the dentist only to those parts that are in need of such treatment, and an antiseptic mouth-wash, together with a proper brush, tooth-powder, or paste, and other utensils for mouth toilet, is all the patient should use. A prescription for a tooth-paste is here given:

| | | |
|---|---|---|
| ℞—Calcii carbonatis ppt., | ℥iijss | |
| Saponis, | ℥ss | |
| Sodii benzoatis, | ʒj | |
| Eucalyptolis, | | |
| Olei menthae pip., | āā m x | |
| Thymolis, | gr. iv | |
| Saccharini, | gr. iij | |
| Glycerini, | q. s. ad pasta. | M. |

Sig.—Use as a tooth-paste.

Any known antiseptic solution may be prescribed. It is as unnecessary as it is unwise for the dentist to prescribe the "cure-all" mouth-washes or those the constituents of which are to him unknown. The official mouth-wash may be prescribed, and diluted to suit the case, a prescription for which follows:

℞—Liquoris antiseptici, f℥viij

SIG.—Dilute with one-half warm water and use as an antiseptic mouth-wash.

While it is unnecessary to remember the formula for this antiseptic solution, it is well to remember that it contains about 2 per cent. of boric acid, 0.1 per cent. of benzoic acid and thymol, 25 per cent. of alcohol, and other aromatics and antiseptics added to water as a vehicle. This prescription illustrates the value of a working knowledge of the pharmacopeia.

In those cases complicated by indigestion and where the patient complains of a sour taste in the mouth, especially upon rising in the morning, due either to local fermentation or to the regurgitation of acid from the stomach, the condition may be corrected by the following prescription:

℞—Sodii bicarbonatis, ʒj

Infusi gentianae comp., f℥iij. M.

SIG.—Take a tablespoonful before meals and on retiring.

When the sour taste comes purely from local fermentation, all that is necessary is to use a warm solution of sodium bicarbonate, or the following alkaline and antiseptic mouth-wash may be presribed:

℞—Sodii bicarbonatis,

Sodii boratis, āā ʒss

Thymolis,

Mentholis, āā gr. ss

Alcoholis, fʒijss

Glycerini, fʒij

Aquae cinnamom, q. s. ad f℥viij. M.

SIG.—Use as an alkaline and antiseptic mouth-wash.

It will be found that either the above solution or the warm sodium bicarbonate solution will correct that transitory sensitiveness which frequently follows the usual treatment. In my own practice I moisten the pumice for polishing with this alkaline and antiseptic solution.—*Cosmos.*

